# Supplementary material for: Quinolizidines as Novel SARS-CoV-2 Entry Inhibitors
Source: Int J Mol Sci. 2022 Aug 25;23(17):9659. doi: 10.3390/ijms23179659 (PMC9455918; doi:10.3390/ijms23179659)

Supporting Information

Title: Quinolizidines as Novel SARS-CoV-2 Entry Inhibitors.

Li Huang, Lei Zhu, Hua Xie, Jeffery Shawn Goodwin, Tanu Rana, Lan Xie, and Chin-Ho Chen *

General methods and chemistry

Reagents were purchased from MilliporeSigma if unspecified. Biotage Initiator (Biotage) was used for microwave heating in synthesis. Silica gel chromatography was carried out on an ISCO CombiFlash Rf flash chromatograph system with a pre-packed Redi Sep Rf Si gel column (Teledyne ISCO) and mobile phase of EtOAc/MeOH/NH_4OH in gradient of increased polarity. Compounds were purified with HPLC using a Varian ProStar HPLC system with a PDA detector and Agilent Zorbax C18 columns (5 µm particle size, 4.6 × 250 mm or 9.4 × 250 mm). The mobile phase used for the HPLC was ACN/MeOH/H_2O/TFA in a gradient of decreasing polarity. All synthesized compounds were confirmed to have purity over 95% by HPLC. Synthesized aloperine derivatives were analyzed on a Shimadzu LC-MS-2020 mass spectrometer with electrospray ionization. ^{1}H NMR and ^{13}C NMR spectra were measured on a Varian 400 MHz or/and 800 MHz spectrometer in methanol-d_4 with Me_4Si (TMS) as internal standard.

The synthesis of compound 9 was accomplished with same method for compound 5 as described in previous report [16].

***N*-[4-(N^{12}-aloperine-yl)butyl]-4-fluorobenzylamine (9):** ^{1}H NMR (800 MHz) (CD_3OD) δ 7.34 (dd, 2H, *J* = 8.4, 5.5 Hz), 7.00-7.06 (m, 2H), 5.54 (bs, 1H), 3.71 (s, 1H), 3.46 (q, 2H, *J* = 13.5 Hz), 2.96-2.99 (m, 1H), 2.39-2.91 (m, 7H), 1.89-2.17 (m, 8H), 1.79 (m, 1H), 1.26-1.69 (m, 13H). ^{13}C NMR (800 MHz) δ 164.0, 162.8, 137.0, 131.7, 131.4, 128.2, 116.1, 115.9, 65.7, 58.6, 56.8, 54.5, 54.3, 53.7, 53.3, 49.9, 36.7, 34.6, 34.5, 30.5, 30.5, 28.6, 26.5, 25.9, 24.5, 23.7. Calcd for $C_{26}H_{38}FN_3$ $(M+H)^+$: 412.3. Found: 412.3.

Figure S1: 1H NMR of compound 9

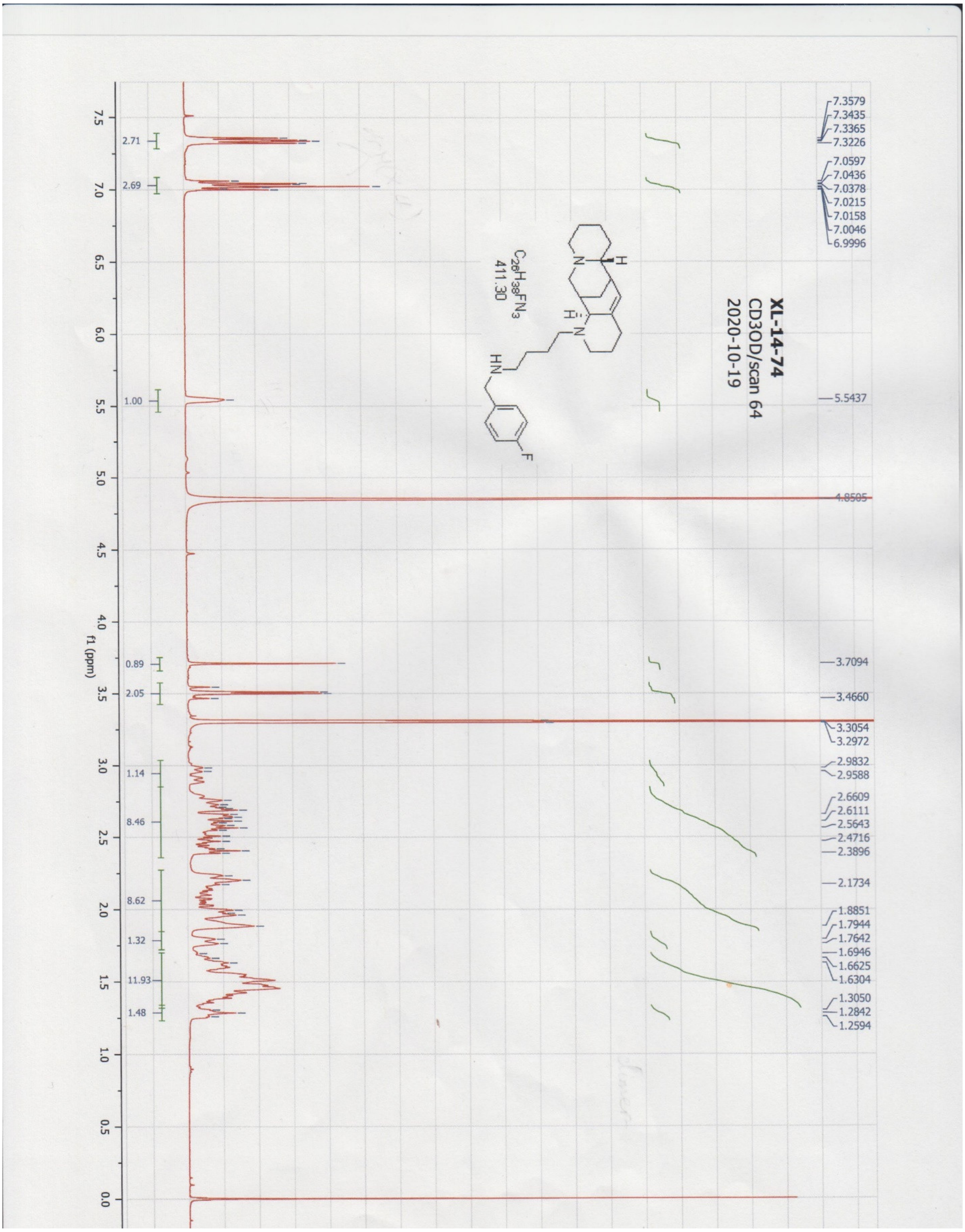

Figure S2: 13C NMR of compound 9

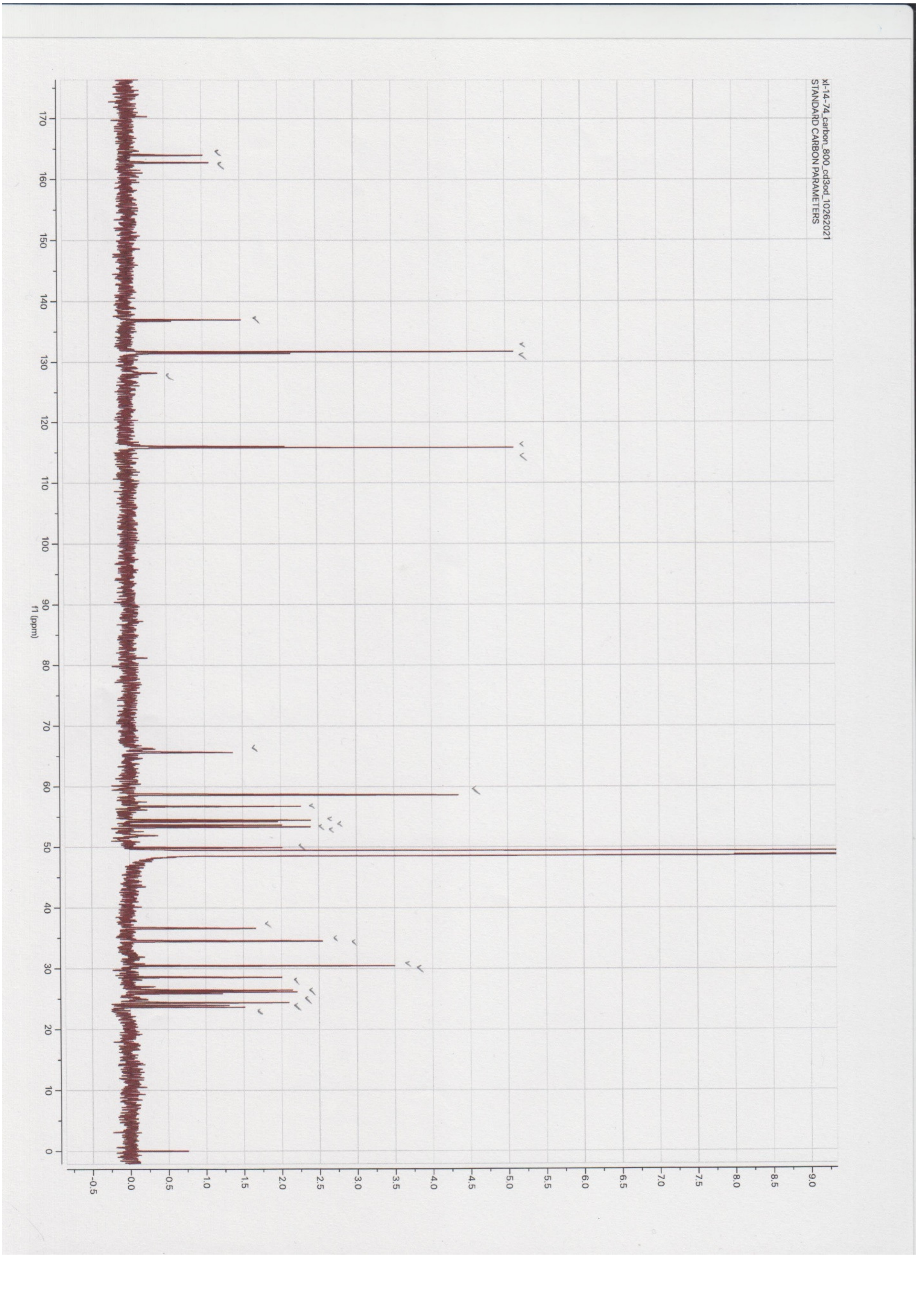

Supplement: Supplementary file 1 [file ijms-23-09659-s001.zip › ijms-1820441-supplementary.pdf]
